# Consumer and Health Care Professional Perspectives on a Digital Platform to Support Preventive Antenatal Care: Qualitative Descriptive Study

**DOI:** 10.2196/98438

**Published:** 2026-08-31

**Authors:** Emma Doherty, Sophie Dilworth, Alice Rice, Taya Wedesweiler, Ranju Shrestha, Sharni Greenwood, Michelle Foster, Janaya Lewis, Justine Daly, Rebecca Wyse, Carly Mallise, Melanie Kingsland

**Affiliations:** 1 Population Health Hunter New England Local Health District Wallsend, New South Wales Australia; 2 School of Medicine and Public Health College of Health, Medicine and Wellbeing University of Newcastle Callaghan, New South Wales Australia; 3 Population Health Research Group Hunter Medical Research Institute New Lambton Heights, New South Wales Australia; 4 Nursing and Midwifery Research Centre Hunter New England Local Health District Newcastle, New South Wales Australia; 5 Single Digital Patient Record Implementation Authority New South Wales Department of Health Chatswood, New South Wales Australia; 6 Women's Health and Maternity Network Hunter New England Local Health District New Lambton Heights, New South Wales Australia

**Keywords:** antenatal care, digital health, preventive care, modifiable health risks, consumers, health care professionals, qualitative research

## Abstract

**Background:**

Modifiable health risks in pregnancy are common and contribute to adverse maternal and infant outcomes, yet preventive care addressing these risks can be difficult to deliver within time-limited antenatal appointments. Hybrid models combining in-person care with digital platforms offer a promising solution; however, there is limited evidence regarding consumer and health care professional perspectives on the preferred content and functionality of such platforms to inform their design.

**Objective:**

This study aimed to explore consumer and health care professional perspectives on the ideal content and functionality of a digital platform to support preventive antenatal care.

**Methods:**

A descriptive qualitative study was conducted across 2 regional maternity services in New South Wales, Australia. Four workshops were held with recently pregnant consumers and antenatal health care professionals, using purposive sampling to include Aboriginal and culturally and linguistically diverse participants. Data were analyzed using applied thematic analysis, guided by a predefined framework, which examined what content should be included, how the platform should function, and why it was needed.

**Results:**

A total of 23 consumers and 12 health care professionals participated. Participants identified key content requirements, including digital risk assessments, evidence-based and personalized information, practical resources, clear support options, and content extending into the postbirth period. Preferred functionalities included early access to the platform, appointment-aligned reminders, integration with routine workflows, tracking tools, multiple content delivery formats, filtering options, and information-sharing options. Participants described gaps in early pregnancy information and noted that digitally provided care could reduce stigma, support more honest disclosure, and enhance communication.

**Conclusions:**

Consumers and health care professionals identified key content and functionality requirements for a digital platform to support preventive antenatal care. Prioritizing credible information, early access, and integration with routine workflows may improve the acceptability and utility of digital solutions. Future research is needed to examine real-world implementation.

## Introduction

Addressing modifiable health risks in pregnancy is recognized as a public health priority [1-3]. Smoking, alcohol consumption, gestational weight gain outside of recommended ranges, poor dietary intake, and physical inactivity are well-established risk factors that adversely affect obstetric outcomes and have lasting consequences for maternal and child health [4-8]. More than 90% of pregnant people experience at least 1 modifiable health risk, with half reporting multiple risks simultaneously [9,10].

Antenatal guidelines recommend addressing modifiable health risks routinely during antenatal visits. Recommended care includes risk assessment, brief advice, and referral to further support, such as health coaching or specialist services [11-14]. Although antenatal health care professionals are well positioned to provide such care, they face challenges in doing so within the constraints of busy, time-limited appointments [15]. Hybrid care models, which combine in-person care with digital platforms, have emerged as a potential solution for overcoming these barriers. Evidence suggests that hybrid care models improve flexibility for patients and allow health care professionals to use limited appointment time for more targeted support, leading to better outcomes [16]. This approach has shown promise in pregnancy, with studies of pregnant people who smoked demonstrating that those receiving hybrid care were substantially more likely to access cessation support and achieve 30-day abstinence than those receiving standard face-to-face antenatal care [17,18].

Digital health strategies [19-22] and evidence-based frameworks for digital health design [23,24] recommend active end-user engagement to maximize acceptability, usability, and effectiveness. However, few digital solutions for preventive antenatal care have been developed with consumers and health care professionals involved from the outset, and studies exploring user preferences have typically examined these groups separately. For example, the US StartSmart project engaged consumers and health care professionals in developing a mobile health intervention to support antenatal care, including screening, brief intervention, and referral [25]. However, user involvement occurred largely after expert-led prototype development, limiting opportunities to shape foundational design decisions. Studies exploring consumer perspectives during the design and evaluation of digital platforms have highlighted the need for credible, locally relevant, and tailored information and support; self-management tools such as tracking, personalized feedback, and planning aids; and simple, intuitive interfaces that facilitate engagement [25-34]. In contrast, studies examining health care professional perspectives have emphasized the value of preappointment risk assessments, evidence-based and guideline-aligned content, tailored risk summaries and counseling prompts, integration with existing workflows and electronic health records, and features that facilitate communication between patients and health care professionals [18,25,26,35-37].

Despite growing evidence on what consumers and health care professionals value in digital tools, these perspectives have rarely been considered together to inform the design of digital platforms for preventive antenatal care. Consequently, there is limited evidence regarding the content and functionality requirements needed to meet the needs of both consumers and health care professionals, which risks the development of solutions that are less acceptable, feasible, and effective within hybrid care models. To address this gap, this study examined and integrated the perspectives of consumers and antenatal health care professionals to identify the core content and functional requirements for a digital platform to support preventive antenatal care.

## Methods

This study is reported in accordance with the COREQ (Consolidated Criteria for Reporting Qualitative Research) guidelines [38].

### Design and Setting

A qualitative descriptive study was conducted during May 2024 and June 2024. Data were collected through workshops with a sample of consumers (recently pregnant people) and antenatal health care professionals from 2 regional maternity services within a local health district in New South Wales (NSW), Australia. Separate workshops were held with consumers and health care professionals at each site, resulting in 4 workshops in total. Workshops were selected as they enabled in-depth exploration and idea generation to pragmatically inform preferences for the content and functionality of a digital platform to support preventive antenatal care. Consumers and health care professionals were separated to minimize power dynamics and encourage open discussion, as consumers may have been less willing to share their honest views in the presence of health care professionals.

### Sampling and Recruitment

Purposive sampling was used to select participants for all workshops. This sampling technique allows the sample to be matched to the aims of the research and is considered appropriate for studies using small samples from limited geographic areas or restricted population definitions [39,40]. Recruitment intentionally targeted Aboriginal and culturally and linguistically diverse consumers, reflecting the population served by participating services and supporting equity considerations in digital health design. Eligible health care professionals included those delivering or supporting antenatal care, with efforts made to include a mix of clinical roles.

Consumer leads (female community members who were part of the study team) distributed information through local networks and community channels, including private social media pages, local cafés, mothers’ groups, and childcare centers. Health care professionals were recruited via emails circulated by maternity service managers. Both groups were given 4 weeks to review the information and contact the study team to indicate interest. All eligible participants who expressed interest were invited to attend and provided written consent upon arrival to the workshops.

### Data Collection

Workshops were facilitated by an experienced female qualitative researcher (SD), supported by a program manager (MK), 1 to 3 project officers (at least 1 of whom identified as Aboriginal; AR, TW, and SG), and 2 consumer leads (RS). The consumer leads were the only study team members with preexisting relationships with participants.

Consumer workshops were held in community venues (with children and carers present), and health care professional workshops were held at maternity service sites. Workshops ran for 2 to 2.5 hours and followed a consistent schedule: introductions, establishment of group expectations, background information on preventive antenatal care, and a series of structured activities (Multimedia Appendix 1). All workshops were audio-recorded, and observation notes were taken to capture contextual factors.

### Data Analysis

Data generated through structured workshop activities (collected on Post-it notes and Kraft paper summaries), together with audio recordings and observation notes, were synthesized into workshop summaries by 1 team member and checked by a second. Accuracy and credibility were supported through review of these summaries by the study’s consumer leads.

Data were analyzed using applied thematic analysis [41]. A predefined “what, how, why” framework derived from the digital health literature was used to examine what content should be included, how the platform should function, and why it was needed [27]. This framework served as the coding structure for organizing and interpreting the data. Two team members independently coded the workshop summaries and met regularly to resolve discrepancies and refine interpretations. Themes were identified iteratively within and across workshops and subsequently synthesized to identify overarching patterns across consumer and health care professional groups.

### Ethical Considerations

The study was approved by the Hunter New England Human Research Ethics Committee (2019/ETH00993 and 2019/ETH00998), the University of Newcastle Human Research Ethics Committee (H-2016-0422 and H-2017-0032), and the Aboriginal Health and Medical Research Council (1236/16). All participants provided informed written consent prior to taking part in the workshops. Data were deidentified prior to analysis, and no identifying information was retained; all audio and written files were stored securely in accordance with institutional privacy requirements. Consumer participants received a gift certificate in recognition of their time, consistent with the NSW Hunter New England Health consumer remuneration rate [42].

## Results

### Participants

A total of 23 consumers participated across 2 workshops (n=13, 57% and n=10, 43%). Their ages ranged from 19 to 39 years. Of the 23 consumers, 6 (26%) identified as Aboriginal, 9 (39%) reported a cultural background other than Australian, and 8 (35%) spoke a language other than English. In addition, 12 health care professionals participated, with 6 (50%) in each workshop, including 5 (42%) midwives, 2 (17%) antenatal clinic managers, 1 (8%) social worker, 1 (8%) obstetrician, 1 (8%) Aboriginal Health Worker, and 2 (17%) students (nursing or midwifery and social work). Only 17% (2/12) of health care professionals identified as Aboriginal.

### Ideal Digital Platform to Support Preventive Antenatal Care

**Figure 1:**
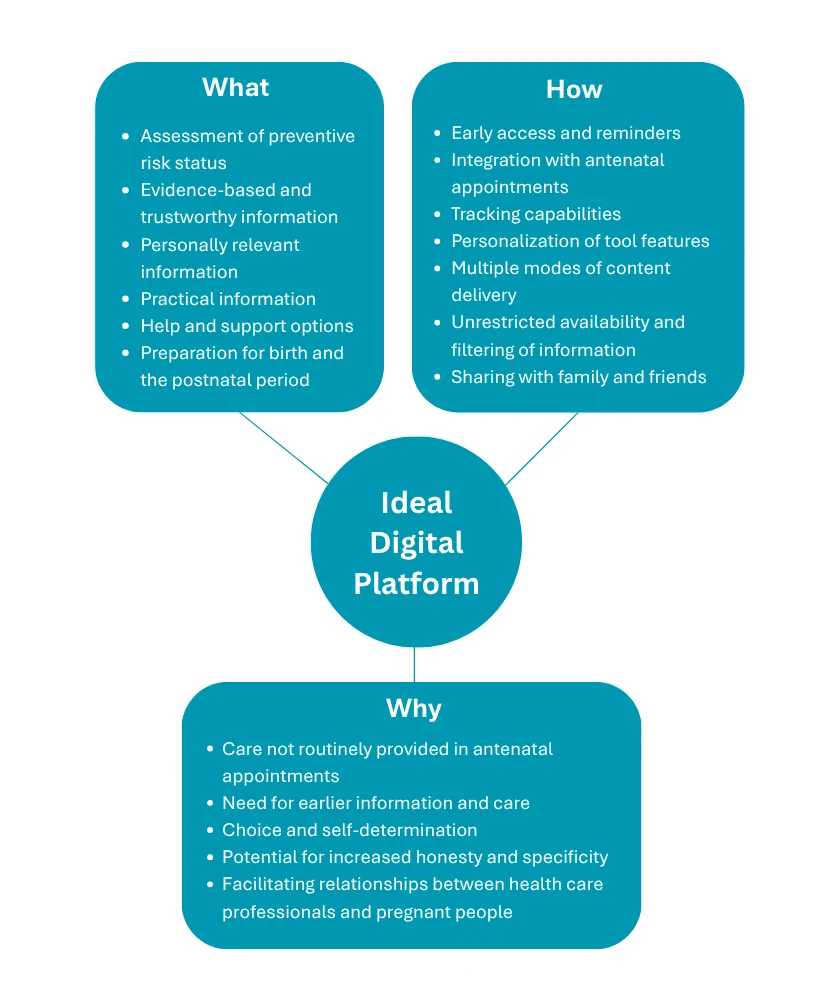
Consumer and health care professional perspectives on the ideal digital platform to support preventive antenatal care within a hybrid model. Hybrid antenatal care refers to the delivery of guideline-recommended preventive care through a combination of a digital platform and face-to-face antenatal visits. In this model, the digital platform supports risk assessment, education, linkage to support, and self-management between appointments, while in-person visits focus on tailored follow-up. This approach differs from standard antenatal care, in which preventive care activities are delivered primarily during face-to-face appointments, with limited structured support between visits.

Guided by the “what, how, why” framework, analysis identified themes relating to the content, functionality, and value of a digital platform to support preventive antenatal care within a hybrid model. Figure 1 and the following sections present these findings from consumer and health care professional perspectives.

### What: Desired Content

#### Assessment of Preventive Risk Status

Participants wanted the platform to collect risk information normally gathered during antenatal visits. Consumers valued having time to “reflect more carefully,” while health care professionals felt digital completion could “increase assessment rates” and “reduce time spent on assessments that take up so much time in appointments.” Both groups wanted clear explanations of upcoming assessments and how responses would be used, with several participants emphasizing the importance of knowing “whether answers form the basis of any mandatory reporting,” particularly when disclosing sensitive information such as alcohol use in pregnancy.

#### Evidence-Based and Trustworthy Information

Participants emphasized the need for reliable, current, and unambiguous guidance, particularly given conflicting online advice. Health care professionals described how “many pregnant people do not know what they can and cannot be doing” and expressed frustration that “everyone is learning things off TikTok.” Both groups wanted clear explanations of mechanisms of harm, such as “what smoking does to the placenta to cause low birth weight,” and clarity on issues such as the harms of vaping and alcohol use. One consumer highlighted the importance of this, noting that “you think that the baby is OK because they look alright, but what people often do not understand is that alcohol effects are usually on brain development rather than appearance.”

#### Personally Relevant Information

Participants wanted information tailored to gestational stage, pregnancy symptoms, and cultural context. One consumer explained that they wanted content about physical activity “matched to what part of pregnancy you are up to and the kinds of pains you could be having.” Health care professionals emphasized cultural inclusivity, noting the need to “tailor it to the girls, who they are, and where they come from,” including culturally specific cooking practices when presenting dietary information and acknowledging differing cultural expectations around physical activity during pregnancy.

#### Practical Information

Participants valued concrete, actionable guidance that could be easily incorporated into daily routines. They wanted recipes (including for special diets), meal planning ideas, strategies for increasing iron intake, and alternatives to alcohol, such as mocktail recipes. One consumer noted that “you are going to need it all when trying to make behaviour changes.” Health care professionals highlighted the value of simple solutions to common issues, such as pregnancy discomforts, explaining that “simple exercise actually makes all those pains better.”

#### Help and Support Options

Participants wanted clear descriptions of available support services and the ability to self-refer directly through the platform. One consumer explained that “if they want referral to Quitline or Get Healthy in Pregnancy, they should be able to just press a button.” Health care professionals felt this approach would help pregnant people choose services that suited them, noting that “not all people will want the same services.”

#### Preparation for Birth and the Postnatal Period

Participants wanted comprehensive guidance on labor, birth, and early postnatal recovery, including practical strategies and clear explanations to help them feel prepared. Health care professionals highlighted gaps in current care, noting that “we really lack anything for that period here.” Participants also wanted information on infant feeding, mental health, and addressing modifiable health risks in the long term, with 1 consumer explaining, “I think it would be great if this app is not just something we use during pregnancy, but also after pregnancy as well.”

### How: Preferred Functionalities and Delivery Features

#### Early Access and Reminders

Participants wanted access to the platform early in pregnancy, ideally at pregnancy confirmation. Health care professionals suggested that “GPs [General Practitioners] could recommend the platform when the pregnancy is confirmed so women can get in early and know what is coming up,” and consumers wanted reminders aligned with upcoming antenatal visits, such as “a week before, then again a couple of days before,” to support completion of digital assessments. Health care professionals described this functionality as a “pre-appointment checklist” that would help pregnant people arrive informed and ready to discuss their priorities.

#### Integration With Antenatal Appointments

Both groups emphasized the need for integration with electronic health records to avoid duplication and ensure information flowed directly to support care. Health care professionals wanted key data visible within their usual workflow, explaining, “I do not want to sit with another app open, it would be ideal if that is already integrated into whatever record keeping system I am using.” They also wanted the platform to identify “red flags” so that important issues were not missed. Consumers highlighted the value of having a space within the platform to record notes and questions between appointments, noting, “I would think of things that I need to ask and then the appointment would come and now I forget.”

#### Tracking Capabilities

Participants valued tracking tools as a way to support self-management and motivation. Health care professionals felt these features could help pregnant people “have that power to self-manage, and say yeah I am doing a good job.” Participants also wanted the platform to integrate with existing health applications commonly used so that information such as daily steps, workouts, or nutrition data could be captured automatically rather than being manually entered.

#### Personalization of Tool Features

Participants wanted personalized profiles, such as due dates and appointment schedules, and opt-in or opt-out features so they could reduce pressure and engage only with the risk factors they felt ready to address. They also saw personalization as a way to support clearer communication and reduce misunderstandings. As 1 health care professional explained, “I might be able to translate ‘do you smoke’ and actually offend someone with the way I asked it,” highlighting how features that allow tailored wording and culturally sensitive phrasing could help avoid miscommunication.

#### Multiple Modes of Content Delivery

Participants wanted information delivered through multiple formats to accommodate different learning styles and literacy levels, including videos, images, quizzes, frequently asked questions, links to reputable external websites, and concise summaries with options to “extend and read more.” Videos were seen as particularly helpful for people who “cannot read or cannot read well.”

#### Unrestricted Availability and Filtering of Information

Participants wanted unrestricted access to all information with filtering and search options for specific needs. Some emphasized the importance of anonymous access to information for sensitive topics they may not want to disclose, such as alcohol use, explaining they wanted to “read about risk factors in their own time.”

#### Sharing With Family and Friends

Participants wanted shareable content to help navigate conflicting advice and involve partners in behavior change. Health care professionals described situations where “their mothers are telling them to do this, and we are telling them something different,” and felt that shareable content could help bridge these gaps. Participants also wanted partners, particularly those who smoke, to have access to relevant information so they could support behavior changes and use strategies to reduce the harm of household exposure.

### Why: Rationale for a Digital Platform

#### Care Not Routinely Provided in Antenatal Appointments

Participants described how time pressures and competing priorities limited preventive care discussions, with health care professionals noting they were “so busy doing all the assessments that we do not get to the actual business end of things.” A digital platform was seen as a way to ensure pregnant people still received essential information “in a safe place, on their phone, at home” when health care professionals could not cover everything in person. Health care professionals felt it could support “a multi-disciplinary, holistic care service” and fill gaps in routine care.

#### Need for Earlier Information and Care

Participants emphasized the long period between pregnancy confirmation and the first antenatal visit, describing feeling unsupported and “going blind for the first four months.” Health care professionals highlighted missed opportunities for early intervention, explaining that “women come in at 19-20 weeks and we have missed so many opportunities for intervening...that information needs to get to women earlier.” A digital platform was viewed as a way to provide timely, evidence-based guidance, with 1 health care professional noting that women would receive “more evidence-based information from this app than googling, which is what they have to do at the moment.”

#### Choice and Self-Determination

Participants valued the platform’s potential to support autonomy and self-directed behavior change. Health care professionals described it as “woman centred,” giving pregnant people “the option and choice in how they engage in their care.” Completing assessments independently was seen as empowering, with 1 health care professional noting that “there will be more drive for change when they are identifying their own needs,” and participants felt that self-initiated referrals would be more effective than health care professional-initiated ones: “if they do the referrals themselves, they are more likely to take them up and engage.”

#### Potential for Increased Honesty and Specificity

Participants believed digital assessments could reduce stigma and support more accurate disclosure of sensitive behaviors. Health care professionals anticipated “more honest and accurate responses,” as digital completion allowed pregnant people to “think about their answer, rather than feeling on the spot and judged.” They also felt that digital formats would “allow women to be more specific,” revealing details often missed in face-to-face discussions.

#### Facilitating Relationships Between Health Care Professionals and Pregnant People

Participants viewed the platform as enhancing, rather than replacing, relationships between pregnant people and health care professionals. Having information in advance was seen as helping health care professionals “review the questions before they come in and arrange the support women need,” while reducing the burden on pregnant people to “retell their story every time they come in.” Health care professionals felt that private access to information could reduce feelings of judgment and “really complement the care we provide and open up discussions,” particularly for emerging issues, such as vaping.

## Discussion

### Principal Findings

This study identified the content and functionality that consumers and health care professionals consider essential for a digital platform designed to support preventive antenatal care. Participants described core content requirements, including structured risk assessments, evidence-based guidance, personally relevant and practical information, clear support options, and preparation for birth and the postnatal period. They also identified preferred delivery features, including personalized, integrated, and multimodal digital functions. Participants further articulated why such a platform is needed, highlighting gaps in standard face-to-face antenatal care; limited support in early pregnancy; and the potential for digital formats to enhance disclosure, autonomy, and communication.

### Comparison With Previous Literature

#### Content

Findings regarding desired content confirm and extend existing research. Consistent with international and Australian studies [18,35,36], participants emphasized the value of structured digital risk assessments, describing them as opportunities for reflection and honesty, while health care professionals highlighted their potential to improve efficiency and comprehensiveness. Participants also wanted reputable, clearly articulated, personalized, and practical information that explains both the harms and benefits associated with modifiable health risks, aligning with previous research. Our findings extend this literature by identifying specific content examples valued by users, including explanations of mechanisms of harm associated with smoking, culturally specific cooking practices, and precise guidance on vaping and secondhand smoke exposure. Participants also emphasized the importance of embedding clear and accessible help and support pathways within the platform, extending previous studies on local support options [29].

These content preferences align closely with behavior change techniques identified in systematic reviews as effective for improving outcomes related to smoking, alcohol, diet, and physical activity [33,43-45], and map directly onto strategies embedded within digital health intervention taxonomies [46], indicating a strong convergence between what consumers and health care professionals consider useful and the approaches most likely to support behavior change. Together, these content areas provide specific, actionable guidance for designing digital platforms that better meet the needs of both pregnant people and health care professionals within hybrid models of preventive care.

#### Functionalities and Delivery Features

Participants also identified functionalities that could maximize usability and integration into antenatal care. Consistent with prior studies [27,29,32,34], participants valued health tracking tools and multimedia formats for supporting self-management and engagement. Our findings extend this evidence by highlighting the importance of early linkage to a digital platform, appointment-aligned reminders, the ability to share information with family and friends, and opt-in or opt-out controls to tailor use. These preferences align with systematic reviews identifying proactive messaging, self-monitoring, feedback, and social support as mechanisms that enhance digital health engagement [27,43-45]. Health care professionals reinforced existing evidence that tailored risk summaries, counseling prompts, and integration with electronic health records are essential for efficiency [25] and further emphasized the value of receiving assessment results in advance to support the prioritization and tailoring of care and multidisciplinary coordination. These findings suggest that digital platforms have the potential to address longstanding challenges in antenatal care, including time constraints, fragmented information flow, and limited opportunities for personalized support, if they include functionalities that enable efficient preparation, more targeted conversations, and more consistent follow-up.

#### Rationale for a Digital Platform

Participants described a persistent gap between pregnancy confirmation and antenatal care, a period marked by limited structured support and missed opportunities for early guidance [47]. Digital platforms were viewed as a practical way to provide consistent information and referral pathways during this time, reducing reliance on informal advice [48]. Participants also felt that digital assessments could support more honest disclosure of sensitive risk factors, echoing evidence that computer-based tools reduce social desirability bias and improve reporting of alcohol, smoking, and substance use [49]. Rather than replacing interpersonal care, digital tools were seen as enhancing communication and cultural responsiveness by helping pregnant people prepare for appointments and engage more confidently in discussions, consistent with research on digital resources supporting communication and self-advocacy [50]. Overall, these perspectives suggest that digital platforms may strengthen preventive antenatal care, with future testing of implementation required to explore whether these perceived benefits are realized in practice.

### Strengths and Limitations

A key strength of this study is the integration of both consumer and health care professional perspectives, enabling a comprehensive understanding of how digital platforms may support preventive antenatal care. Strong representation from Aboriginal and culturally and linguistically diverse participants helped embed equity considerations within the findings [51]. Workshops conducted in 2 regional maternity services ensured context-specific detail relevant to local workflows and community preferences. Limitations include the regional setting, which may limit transferability, and the developmental nature of the study, meaning that the findings reflect anticipated rather than observed use.

### Conclusions

This study identified the content and functionality that consumers and health care professionals view as important for a digital platform designed to support preventive antenatal care. Future research should examine real-world implementation to determine whether the anticipated benefits of a hybrid approach to preventive antenatal care are realized.

## Data Availability

The datasets generated and analyzed during this study are available from the corresponding author upon reasonable request.

## Appendix

Multimedia Appendix 1 Workshop activities guide.

## Abbreviations

COREQ: Consolidated Criteria for Reporting Qualitative Research
GP: General Practitioner
NSW: New South Wales
