# Supplementary material for: Consumer and Health Care Professional Perspectives on a Digital Platform to Support Preventive Antenatal Care: Qualitative Descriptive Study
Source: JMIR Form Res. 2026 Aug 31;10:e98438. doi: 10.2196/98438 (PMC13575547; doi:10.2196/98438)
Supplement: Supplementary file 1 [file formative_v10i1e98438_app1.pdf]

Multimedia Appendix 1: Workshop Activities Guide

Consumers Workshops

Activity One: "Blue Sky" brainstorm of ideal digital platform

- Individual activity with ideas written on post-it notes in response to: *"In an ideal world, what would a digital platform that could improve care for smoking, alcohol consumption, weight, nutrition and physical activity look like?"*
- Notes were placed on kraft paper displayed on venue walls, each paper representing a separate risk factor. Non-topic specific ideas were grouped into a general category.

Activity Two: Brainstorm content and functionality

- Small groups focused on either (1) smoking and alcohol, or (2) weight, nutrition and physical activity.
- Discussion prompts included: *"What would make the content of the digital platform relevant to you?"* and *"What digital features and functions would make it work for you and other pregnant people?"*
- A study team member facilitated the discussion and scribed onto kraft paper structured around *what, how, why*.

Activity Three: Whole group synthesis

- Kraft papers from each small group were displayed on venue walls.
- A spokesperson for each group presented a summary of their discussions.
- A whole group discussion was guided by the Facilitator who added further notes to the kraft papers.
- Post-it notes summarising ideas from Activity One were incorporated.
- The importance of each idea was then assessed through group voting by hand raising.

Providers Workshops

Activity One: "Blue Sky" brainstorm of ideal digital platform

- Individual activity with ideas recorded on worksheets structured around ask, advise, refer* in response to*: "In an ideal world, how would a digital platform ensure the best care for smoking, alcohol consumption, weight, nutrition and physical activity is provided to all pregnant people? How could this platform support you to provide care? How could it complement in-appointment care?"*
- Whole group sharing and discussion with scribing by a study team member.

Activity Two: Integration with clinic workflows and processes

- Individual activity with ideas recorded on worksheets in response to: *"How could a digital platform fit into your clinic and work processes? What features and functions would make it work for you in your role? When would it be useful to use or receive information from a woman, and how often?"*
- Whole group sharing and discussion with scribing by a study team member.
